# Hurrying to Death

**Published:** 1872-09

**Authors:** 


### HURRYING TO DEATH.

Very recently a distinguished professor in a Virginia University wanted to deposit a letter in the post-office on the other side of the railroad track. A locomotive was approaching; he thought he could cross before the ponderous engine could come along. He miscalculated the speed. In another moment he was a shapeless mass. Had he waited two minutes, — half a minute, the train would have passed along, and he could have leisurely deposited his letter.

A young lady wished to show her friends how easily she could cross in front of a locomotive ; she did cross, but her streaming dress caught in the passing wheel, drawing her back under its crushing weight.

One afternoon, about sundown, a young wife was looking out of the window of her beautiful country home for the return of her young husband from the city. For the six months of their married life just past they had been busily fixing up their country place; both were young, both healthy ; the husband was in business on his own account, with every prospect of increasing success. She saw him get out of the cars, and passed down-stairs to greet him at the door; but when she reached it, he was not there. She thought he was playing her a little trick; she called for him playfully, affectionately, but there was no answer; she saw a crowd of men approach the gate, open it, come up the path with her dead husband. He did alight from the cars, and safely stepped on the platform before the station. There was a train coming in an opposite direction; he thought he had plenty of time to cross in front of it, and did cross, except by one single inch ; the wheel struck the heel of his boot, wheeled him round under the cars, and all was over; one minute longer, and he could have crossed with the locomotive behind him.

Limbs are broken, lives are lost every year, in any large city, by attempting to cross in front of a moving horse or vehicle; an infatuation seems to come across many under such circumstances, as if they were willing to risk limb and life itself to save one single sixty seconds.

"'TWAS THE NIGHT BEFORE CHRISTMAS,"

And a happy mother and five or six children, in Brooklyn, were looking for the father to come over from his business place in New York, to arrange for that mysterious visitor called Santa Claus, and Kriss Kringle. It was just dark. He was somewhat later than usual, but surely the next boat would bring him over; there it was at the wharf, for they could see it through the window, not a quarter of a mile away. Fast footsteps pattered along, on the pavement, coming nearer, coming opposite, passing along, and dying in the distance; other footsteps approached, passed on, fewer and fewer all the time; and at length there was silence; father had not come, so they thought; but they were mistaken; he did cross in that boat, loaded with the most beautiful assortment of toys and sweetmeats, something even for the baby, just nine months old. In his hurry to meet the happy faces which he very well knew were peering through the darkness at the window pane, he made an effort to step from the boat before she touched the platform; the distance was greater than he supposed; he fell half through into the water, his body caught between the timbers, — and life was gone. Fifteen seconds more, and he could have stepped on solid ground. To save a minute, a life was lost; just as in another direction we waste time and lose eternity; we spend our whole existence here; we hurry on in our own business for the accumulation of money, giving no thought of preparation for the great future which so nearly concerns us all, and in a moment to pass away, without any preparation whatever — so busy in getting gold that there is no time given to save the soul.

